# Regulating Inflammation in the Heart

**Published:** 2007-03

**Authors:** DeLisa Fairweather

**Affiliations:** *Department of Environmental Health Sciences and Pathology, Johns Hopkins University Bloomberg School of Public Health and School of Medicine, Baltimore, USA*

**Keywords:** cytokines, inflammation, innate immunity, myocarditis, tolerance, virus

## Abstract

Cardiovascular disease is the leading cause of death in the United States and estimated to be the leading cause of death worldwide by the year 2020. Many pathogens including bacteria, protozoa, and viruses are associated with inflammatory heart disease in patients, and can induce similar disease in animal models. Recognition of pathogens by the innate immune system leads to the release of proinflammatory cytokines that both reduce infection and increase inflammation in the heart. Signaling pathways that will eventually down-regulate cardiac inflammation, such as anti-inflammatory cytokines and regulatory T cells, are also initiated during the innate immune response. A careful balance between activation and regulation of the immune response to infection reduces the severity of inflammation in the heart, the leading cause of cardiovascular diseases such as atherosclerosis, myocarditis and dilated cardiomyopathy.

## INTRODUCTION

Cardiovascular disease (CVD) is the single largest cause of death in men and women in the United States (1). Inflammation underlies the pathogenesis of many common CVD such as myocardial infarction, atherosclerosis, myocarditis and congestive heart failure (2). Enteroviruses, like coxsackievirus B3 (CVB3), infect vascular tissues in culture and have been detected in atherosclerotic plaques from patients (3-5). Furthermore, CVB3 is a primary etiologic agent leading to inflammation of the myocardium, or myocarditis, which often progresses to dilated cardiomyopathy (DCM) and congestive heart failure (6). While most individuals recover from acute inflammation of the heart, susceptible individuals may develop chronic inflammation associated with fibrosis, DCM and heart failure (6-9). A similar pathological picture is observed by inoculation of susceptible strains of mice with CVB3 or adjuvant and cardiac myosin (reviewed in 10, 11). Our laboratory has used the CVB3-induced model of myocarditis to explore the immune factors responsible for recruiting and regulating inflammation in the heart.

### Increases Inflammation in the Heart

**Figure 1:**
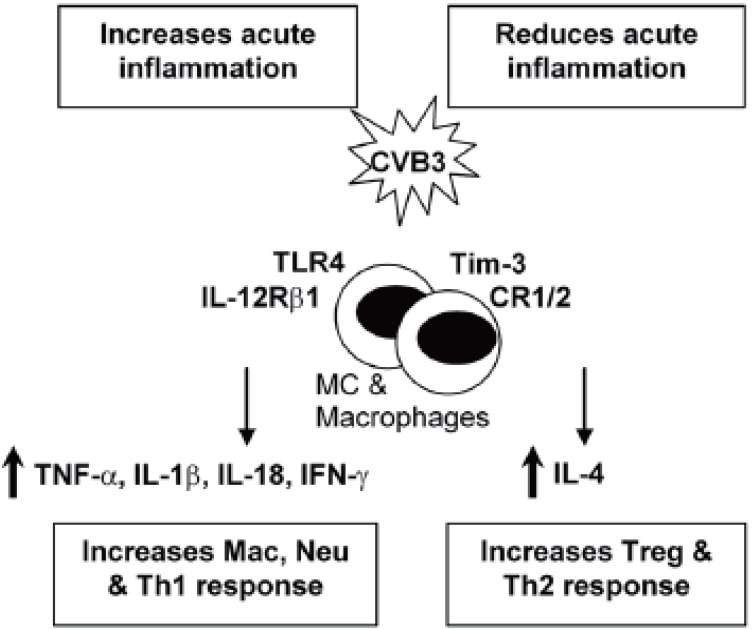
Mechanisms involved in regulating acute inflammation in the heart. CVB3 infection increases TLR4 and Tim-3 expression on macrophages and MC during the innate immune response. TLR4 and IL-12Rβ1 signaling increase IL-1β, IL-18 and IFN-γ levels and inflammation in the heart during acute myocarditis. IFN-γ increases the number of macrophages (Mac) and neutrophils (Neu) in the heart, which help control viral replication. To regulate the Th1-type immune response in the heart, Tim-3 increases CD80/ CTLA-4 expression and Treg cell populations in the heart. Tim-3 and CR1/2 signaling and IL-4, Th2-type immune responses reduce inflammation in the heart and increase Treg populations.

TLR4 and IL-12Rβ1 signaling: An immediate response to pathogens is essential for host defense during the early stages of infection. Innate immune cells, such as macrophages and dendritic cells, recognize pathogen associated molecular patterns (PAMPs) that are unique to particular microorganisms but are either not present in the host or only present following damage to self tissue (8, 12). We have shown that Toll-like receptor TLR4, which recognizes several bacterial, viral and self ligands, is upregulated on macrophages and mast cells (MC) immediately after CVB3 infection, resulting in a burst of proinflammatory cytokines in the heart 6 hours after infection (8, 13). TLR4 deficient mice infected with CVB3 develop significantly reduced acute myocarditis that correlates with reduced interleukin (IL)-1β and IL-18 levels in the heart, indicating that TLR4 signaling increases inflammation in the heart (8, 14). IL-12Rβ1 deficient mice (a receptor for IL-12 and IL-23) develop reduced inflammation, IL-1β and IL-18 in the heart, similar to TLR4 deficient mice, suggesting an overlapping role for these two receptors (14) (Figure 1). Furthermore, TLR4 signaling, IL-1β and IL-18 increase atherosclerosis in the ApoE deficient mouse model (15-17), indicating that similar mechanisms increase inflammation in the myocardium and vasculature.

**Figure 2:**
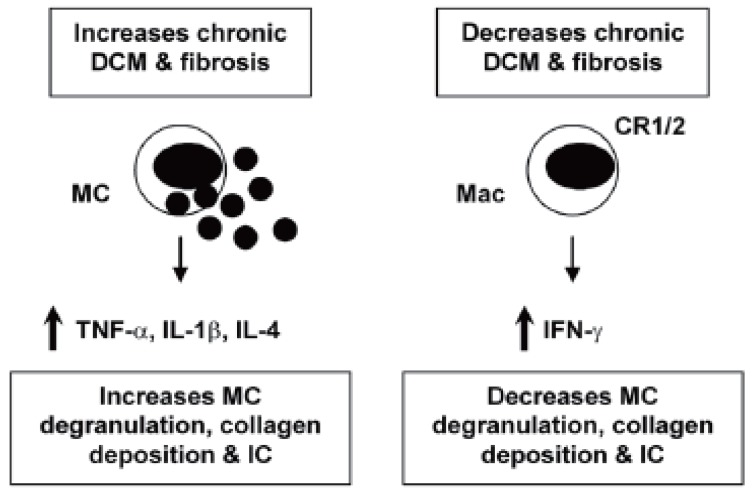
Mechanisms that regulate chronic inflammation, fibrosis and DCM in the heart. Th2-type, IL-4 responses stimulate MC degranulation and release of the profibrotic cytokines TNF-α, IL-1β and IL-4 in the heart. Cardiac fibrosis leads to DCM and congestive heart failure. IFN-γ blocks IL-4 production thereby reducing MC degranulation and fibrosis. CR1/2 on macrophages (Mac) reduces immune complex (IC) deposition in the heart, reducing fibrosis and DCM.

TNF-α and IL-1β: For some time the cytokines tumor necrosis factor (TNF)-α and IL-1β have been known to contribute to heart failure by inducing hypertrophy and/ or apoptosis of cardiac myocytes (9, 18). TNF-α and IL-1β are also key proinflammatory cytokines. Inoculation of mice with TNF-α or IL-1β during the innate immune response to CVB3 infection significantly increases inflammation in the heart (19, 20), indicating the important role the innate immune response plays in modulating adaptive immunity. TNF-α up-regulates MHC class II and the costimulatory molecules CD80 and CD86 on antigen presenting cells following infection or adjuvant treatment (21). Furthermore, myocarditis can be transferred by injection of cardiac myosin-reactive T cells into mice pretreated with TNF-α (22). TNF-α and IL-1β levels are significantly increased in the heart during the innate (6 hours after infection) and adaptive (during acute myocarditis) immune response in susceptible strains of mice (i.e. BALB/c) that develop chronic myocarditis and DCM (13, 23). Thus, TNF-α and IL-1β are involved in the pathogenesis of acute myocarditis and chronic heart disease by increasing inflammation and fibrosis, respectively (Figure 1, 2). Thus, treatments aimed at blocking or reducing these cytokines may produce contradictory results depending on the stage of heart disease.

IFN-γ / Th1-type immune response: IL-12 is potent at inducing IFN-γ production from natural killer and T cells, and promotes the differentiation of naïve T cells to a T helper (Th)1-type phenotype (24). Th1-type immune responses have been implicated in many inflammatory conditions such as colitis, multiple sclerosis, diabetes, and rheumatoid arthritis (25). IL-12 is also required for resistance to viral infections by stimulating TNF-α and IFN-γ production from inflammatory effector cells, which act synergistically to control infection (26). Following CVB3 infection, IFN-γ increases macrophage and neutrophil populations in the heart resulting in reduced viral replication (26) (Figure 1). In contrast, IFN-γ reduces chronic myocarditis by inhibiting myocardial and pericardial fibrosis, DCM and heart failure (7) (Figure 2). The association of many autoimmune diseases with a Th1-type immune response may be explained, in part, by the fact that proinflammatory responses involving TLR4 signaling, IL-1β and IL-18 lead to greater IFN-γ production (27) (Figure 1). In support of this idea, we have found that male mice develop significantly increased inflammation in the heart compared to females, similar to the increased CVD observed in humans (1), and have increased levels of IL-1β, IL-18 and IFN-γ in the heart (28).

### Decreases Inflammation in the Heart

IL-4/ Th2-type immune response: Female mice develop an IL-4, Th2-type immune response following CVB3 infection with significantly lower myocarditis compared to males (28). Although IL-4 increases B cell numbers in the heart of female mice following CVB3 infection, macrophages and neutrophils, which are recruited by IFN-γ, are the predominant infiltrate in the heart during acute myocarditis (26). Thus, overall males develop increased inflammation in the heart following CVB3 infection (28). Although a Th2-type response to CVB3 infection results in reduced acute myocarditis, IL-4 increases chronic heart disease by increasing MC degranulation and fibrosis (7) (Figure 2). Furthermore, mice susceptible to chronic heart disease (i.e. BALB/c) have increased numbers of MC and elevated levels of IL-4 in the heart during the innate and adaptive immune response to CVB3 infection compared to C57BL/6 mice (13, 23). MC express TLR4 and produce a rapid burst of cytokines when stimulated, including TNF-α IL-1β and IL-4 -all cytokines associated with increased fibrosis (7, 13, 29) (Figure 2). Mice deficient in IFN-γ (with increased IL-4 levels) develop increased chronic myocarditis, DCM and heart failure associated with increased MC degranulation, fibrosis and pericarditis (7) (Figure 2). Thus, although IL-4 reduces acute inflammation in the heart by inhibiting Th1-type immune responses (Figure 1), IL-4 contributes to chronic heart disease by increasing fibrosis (Figure 2). This could partly explain why chronic heart disease and congestive heart failure occur more frequently in women (1, 23).

CR1/2 signaling: Complement and complement receptors (CR) play a central role in immune defense by initiating the rapid destruction of invading microorganisms, amplifying the innate and adaptive immune responses and clearing immune complexes (IC). Defects in complement or CR have been associated with the development of ICmediated autoimmune diseases like systemic lupus erythematosus (30, 31). Because of the many roles for complement in protecting against infection and amplifying the immune response, defects in complement or CR could 1) increase myocarditis by allowing increased viral replication, 2) decrease inflammation because of the absence of co-stimulation of innate and adaptive immunity, or 3) increase IC-mediated heart disease by inhibiting clearance of IC. We found that CR1/2 deficiency did not increase CVB3 replication in the heart, but significantly increased CVB3-induced myocarditis, pericardial fibrosis, and IC deposition in the heart resulting in an early progression to DCM and heart failure (32) (Figure 2). Thus, CR1/2 expression is not necessary for effective clearance of CVB3 but important in reducing inflammation and fibrosis associated with IC deposition in the heart. CR1/2 may not have been necessary to clear CVB3 infection because IFN-γ levels were unaffected by CR1/2 deficiency and IFN-γ is critical in inhibiting CVB3 replication (26, 32, 33). CR1/2 deficient mice had increased numbers of macrophages and IL-1β levels in the heart, indicating that CR1/2 signaling also decreases this key proinflammatory response in the heart following viral infection.

Tim-3 signaling & Treg cells: The family of genes encoding T cell immunoglobulin mucin (Tim) proteins has been linked to susceptibility to allergy and autoimmune diseases (34, 35). Tim-3 is highly expressed on Th1 type CD4^+^ T cells, and expressed at low levels on monocyte/macrophages, natural killer cells and MC (36-38). Treatment of mice with antibodies to block Tim-3 receptor signaling increases experimental autoimmune encephalomyelitis, an adjuvant-induced model of multiple sclerosis, and CVB3-induced myocarditis (36, 37). We found that blocking Tim-3 signaling during the innate immune response to CVB3 infection increases the number of macrophages and neutrophils (CD11b+ cells) and reduces regulatory T cell populations (Treg) in the heart during acute myocarditis (36) (Figure 1). Blocking Tim-3 reduces the expression of the co-stimulatory molecule CD80 on MC and macrophages during innate immunity, which is associated with decreased innate expression of CTLA-4, a molecule important for the development of Treg cell populations (36). Treg cells inhibit Th1-type immune responses by releasing anti-inflammatory cytokines such as IL-10 and by inducing apoptosis of inflammatory cells (39). Thus, regulation of inflammation by Tim-3 signaling is initiated during the innate immune response to CVB3 infection and critically influences the severity of acute myocarditis.

## CONCLUSION

Inflammation in the heart can be reduced or possibly even prevented if proinflammatory immune responses are appropriately down-regulated. Receptors such as Tim-3 and CTLA-4, cytokines like IL-4 and IL-10, and specialized cells such as Treg populations, work together to keep Th1-type immune responses in check (Figure 1). Tim-3 is a key regulator of inflammation in the heart, providing inhibitory signals during the innate and adaptive immune response to infection that increase expression of CTLA-4 leading to increased numbers of Treg cells and reduction of acute myocarditis. Although Th1-type responses increase inflammation in the heart (Figure 1), IFN-γ is also critical for reducing viral replication and viral damage to cardiac tissues. Even though IL-4 inhibits Th1-type proinflammatory responses, it also promotes chronic myocarditis and DCM by increasing fibrosis (Figure 2). Thus, proinflammatory and regulatory arms of the immune response to infection must be kept in balance in order to reduce the severity of inflammation in the heart following infection.
